# Development and In Vitro Evaluation of Stearic Acid Phosphotyrosine Amide as New Excipient for Zeta Potential Changing Self-Emulsifying Drug Delivery Systems

**DOI:** 10.1007/s11095-020-02802-2

**Published:** 2020-04-06

**Authors:** Felix Prüfert, Franz Fischer, Christina Leichner, Sergey Zaichik, Andreas Bernkop-Schnürch

**Affiliations:** Department of Pharmaceutical Technology, Institute of Pharmacy, University of Innsbruck, Innrain 80/82, 6020 Innsbruck, Austria

**Keywords:** SEDDS, zeta potential changing drug delivery systems, phosphotyrosine, phosphatase

## Abstract

**Abstract:**

**Purpose:**

Development of zeta potential changing SEDDS containing newly synthesized derivative stearic acid phosphotyrosine amide.

**Methods:**

Stearoyl chloride was conjugated with phosphotyrosine, which is substrate for the brush border enzyme intestinal alkaline phosphate. The synthesized derivative was implemented in different SEDDS formulations and the zeta potential changing properties and the concluding mucus diffusion abilities were evaluated.

**Results:**

Stearic acid phosphotyrosine amide was successfully synthesized and incorporated into SEDDS. A SEDDS formulation containing the new derivative showed a zeta potential of −14 mV before, and + 2 mV after enzymatic cleavage by intestinal alkaline phosphatase. Experiments on a Caco-2 monolayer demonstrated that the phosphate cannot only be cleaved by isolated enzyme, but also by enzyme, which was expressed by cells. The mucus diffusion abilities of the untreated, negatively charged SEDDS were significantly higher compared to the enzymatically cleaved, positively charged SEDDS.

**Conclusion:**

The developed stearic acid phosphotyrosine represents a promising excipient for zeta potential changing SEDDS.

**Figure:**
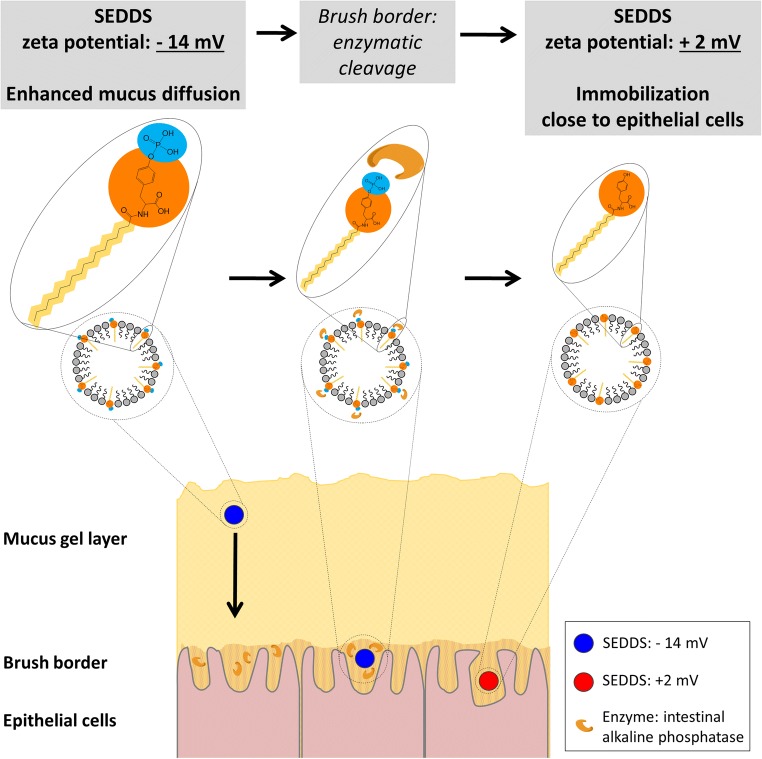
Graphical Abstract

## Introduction

With an increasing availability of macromolecular drugs, such as biologics and gene therapeutics, the demand for efficient oral drug delivery systems is rising. Most patients favor the oral administration route and a high patient compliance is the key to an increased therapeutic effect. However, the oral delivery of such medicines faces several challenges, such as the mucus gel layer covering the epithelial surface (1), enzymatic degradation (2), and minor cellular uptake (3). A promising approach to overcome these challenges are zeta potential changing drug delivery systems – a concept that has been evaluated in different recent publications (4–10). These nanocarriers are equipped with certain side chains that are substrate for membrane-bound intestinal enzymes. After penetration of the mucus layer as negatively charged systems, the enzymatic cleavage of these side chains induces a zeta potential change to positive and thus facilitates the immobilization of the nanocarriers close to the epithelial surface due to ionic interactions with the negatively charged cell surface and mucus layer. Consequently, the cellular uptake is promoted due to the increased residence time and a favored endocytotic uptake of positively charged particles (11,12). So far, polymeric nanoparticles and self-emulsifying drug delivery systems (SEDDS) with zeta-potential changing properties have been investigated. For the present study, SEDDS have been chosen as basis for the nanocarriers: these mixtures of surfactants, oils and solvents form emulsions of lipophilic droplets once they get into contact with intestinal fluids. SEDDS already show good mucus-permeating abilities as well as a high cellular uptake and furthermore can protect their encapsulated drug load from enzymatic degradation (12). These assets can be further improved by the addition of zeta potential changing properties (7,9).

To achieve this, phosphotyrosine - a substrate to intestinal alkaline phosphatase - was conjugated with stearoyl chloride to create a molecule with amphiphilic properties, which allowed for an implementation into SEDDS. Their zeta potential and the change after enzymatic hydrolysis by intestinal alkaline phosphatase was evaluated. With mucus diffusion studies the mucus diffusion capabilities of the untreated, negatively charged SEDDS and the immobilization of the enzymatically cleaved positively charged SEDDS was investigated.

## Materials and Methods

### Materials

Phosphotyrosine was purchased from Bachem Holding AG, Bubendorf, Switzerland. Capmul MCM was donated by ABITEC Corporation, Columbus, OH, USA. IAP (phosphatase, alkaline from bovine intestinal mucosa), phosphatase inhibitor (phosphatase inhibitor cocktail 2) and all other substances were obtained from Sigma-Aldrich Handels GmbH, Vienna, Austria. Cell culture microplates were purchased from Greiner Bio-One GmbH, Kremsmünster, Austria. All other cell culture supplies were purchased at Biochrom GmbH, Berlin, Germany. Porcine intestine was obtained from a local slaughterhouse.

### Synthesis and Characterization of Stearic Acid Phosphotyrosine Amide

Stearic acid phosphotyrosine amide (SA-PTyr) was synthesized by the reaction of the amino group of phosphotyrosine (PTyr) with the acid chloride group of stearoyl chloride. In brief, 1.25 g of stearoyl chloride was heated to 60°C and then mixed with 70 mL of N-methyl-2-pyrrolidone as solvent. Subsequently, 1 g of PTyr in powder form was dissolved in the mixture and the reaction mixture was incubated for 90 h. Afterwards, the product was precipitated by the addition of water. The product was purified from N-methyl-2-pyrrolidone and unconjugated PTyr by repeated washing with water and filtration steps and subsequently desiccated in a drying chamber.

Via spectrophotometry, the amount of yielded product was determined. Hence, samples of the newly synthesized SA-PTyr were dissolved in DMSO in a concentration of 1 mg/mL and the absorbance (λ = 274 nm) was compared to a calibration curve of PTyr standards. The synthesized SA-PTyr was identified by mass spectrometry. A 0.01% (*w*/*v*) solution of SA-PTyr in DMSO was prepared and qualitatively analyzed with a Bruker mikrOTOF – QII mass spectrometer (Bruker Corporation, Billerica, MA, USA).

The HLB value of the synthesized compound was calculated according to Griffin.

### Preparation and Characterization of SEDDS

SEDDS formulations were composed of stearic acid phosphotyrosine amide, Capmul MCM, TWEEN 80 and PEG 400 in different ratios and combinations. The (semi-)solid components were melted before use. An overview of the compositions is provided in Table 1. The excipients for each formulation were then mixed and homogenized at 70°C to obtain a single phase preconcentrate, respectively. For the mucus diffusion studies, moreover 0.1% (*w*/*v*) of fluorescein diacetate (FDA) was added to the preconcentrate of formulation #3 and further mixed and homogenized. Formulation #3 was chosen for this purpose, as it was the most promising one according to our results.

**Table 1:** Compositions of different SEDDS formulations. SA-PTyr was weighed in as solid and melted before the other excipients were added as liquids in stated ratios to reach the given final concentration of SA-PTyr, respectively

**Formulation**		**#1**	**#2**	**#3**	**#4**
Final concentration of:					
		[% (m/v)]	[% (m/v)]	[% (m/v)]	[% (m/v)]
**SA-PTyr**		4	1.8	0.65	0.35
In SEDDS formulation:					
		[% (v/v)]	[% (*v*/v)]	[% (v/v)]	[% (v/v)]
	**Capmul MCM**	10	10	9	10
	**TWEEN 80**	30	30	35	30
	**PEG 400**	60	60	56	60

For characterization and the following studies, emulsions of these SEDDS formulations were prepared. Therefore, the SEDDS preconcentrates were emulsified in a concentration of 1% (*v*/v), respectively, in order to ensure a reliable detection in the following experiments.

#### Investigation of Zeta Potential Change

Based on the results of the SEDDS characterization, suitable SEDDS formulations were selected for investigations of zeta potential change. Therefore, each 1 mL of the SEDDS emulsions were mixed with 2 μL of intestinal alkaline phosphatase solution (1000 U/mL) and incubated at 37°C. The zeta potential before and after the addition of the IAP was determined by electrophoretic light scattering with a Zetasizer Nano ZSP (Malvern Instruments Ltd., Worcestershire, United Kingdom) with a laser wavelength of 633 nm and at a temperature of 37°C. For a selected SEDDS formulation, the kinetics of the change of the zeta potential over time was evaluated at predetermined time points.

### Experiments on Caco-2 Cell Monolayers

The enzymatic cleavability of the SA-PTyr incorporated in SEDDS formulations by Caco-2 cells was investigated:

#### Viability of Caco-2 Cells

In order to evaluate the viability of the Caco-2 cells upon incubation with SEDDS emulsion in the upcoming experiment a resazurin assay was used (13). Caco-2 cells in a concentration of 2 × 10^4^ cells/mL were seeded in 24-well plates and incubated at 37°C and 5% CO_2_ for 14 days with a change of the culture medium every second day. 500 μL of 1% (*v*/v) SEDDS formulation, pure MEM as negative control or Triton X® 100 5% (v/v) as positive control were added to the wells, respectively. The experiments were performed in triplicates. After incubation for 6 h at aforementioned conditions, samples were removed and cells washed twice with 500 μL of PBS. Then, each 500 μL of resazurin solution was added to the wells and after an incubation of 2 h the fluorescence measurements at an excitation wavelength of 540 nm and an emission wavelength of 590 nm were performed.

#### Enzymatic Phosphate Cleavage by Caco-2 Cells

The enzymatic cleavage of conjugated phosphotyrosine present in formulation #3 during incubation on a Caco-2 monolayer was evaluated. Caco-2 cells were grown in a 24-well plate starting with an initial concentration of 2 × 10^4^ cells/mL and incubated at 37°C and 5% CO_2_ for 14 days, changing the culture medium every second day. After a confluent monolayer was established, the cells were washed three times with HBS. 1% (*v*/v) SEDDS emulsions were applied to the cells and incubated at aforementioned conditions for 2 h. At predetermined time points, samples of 50 μL were taken and mixed with 5 μL of 3.6 M H_2_SO_4_ to immediately stop the enzymatic reaction.

To quantify the amount of cleaved phosphate a malachite green assay was employed as described previously (5). Each sample was mixed with 100 μL of malachite green reagent (a mixture of 10 mL of a 0.15% malachite green solution in 3.6 M H_2_SO_4_, with 6 mL of a 7.5% ammonium molybdate solution and 0.4 mL of an 11% Triton-X 100 solution). By subsequent measurement of the absorbance of the samples and a calibration curve of phosphate standards at a wavelength of 630 nm the amount of cleaved phosphate was determined.

### Mucus Diffusion Studies

In order to investigate the diffusion of a selected SEDDS formulation through mucus, two different mucus diffusion studies were performed. Based on results of preceding experiments, formulation #3 was chosen for this purpose. Mucus was obtained from porcine intestine. Therefore, freshly excised small intestine was split into segments and cut open longitudinally and then the mucus was carefully scraped off the tissue and subsequently purified and homogenized by mixing with sodium chloride solution (0.1 M). Afterwards, it was centrifuged at 13,000 *g* and 4°C for 2 h. The supernatant and granular material at the bottom were discarded. The homogenized mucus was used for the mucus diffusion studies as described below.

For both diffusion study setups, samples of 1% (*v*/v) SEDDS emulsions of formulation #3 labelled with 0.1% of the lipophilic fluorescence marker fluorescein diacetate (FDA) were used (14). Therefore, 0.1% (*w*/*v*) of fluorescein diacetate (FDA) was added to the SEDDS preconcentrate of formulation #3 and sonicated for 10 min. Further homogenization and preparation of the FDA labelled SEDDS emulsion was done as described in section 2.3.

In order to investigate the differences in mucus diffusion between SEDDS before and after enzymatic cleavage and thus zeta potential change, the SEDDS emulsions were pretreated by incubation without and with the presence of IAP for 2 h at 37°C under gentle shaking at 300 rpm, to obtain samples with negative and positive zeta potential, respectively. In order to suppress potential enzymatic activity of the collected mucus during the diffusion study that could lead to undetected additional enzymatic cleavage and thus change of zeta potential, phosphatase inhibitor was added to the samples without IAP to prevent a potential change of zeta potential, but to maintain the negative zeta potential. Aliquots of these samples were then used for the mucus diffusion studies in the transwell chambers and the rotating tube, respectively. The setups of both studies are described in the following sections (2.5.1 and 2.5.2).

#### Transwell Chambers

A transwell chamber setup (14) was used to investigate the amount of SEDDS migrating from the donor compartment through the mucus layer to the acceptor compartment. ThinCert™ inserts (Greiner Bio-One GmbH, Kremsmünster, Austria) with a pore diameter of 3.0 μm and a culture surface of 33.6 mm^2^ were covered each with 50 mg of mucus and placed in 24-well plates. The donor compartments were filled with 250 μL of the samples (SEDDS emulsions of FDA labelled formulation #3, see section 2.5) and the acceptor compartments with 500 μL HBS, respectively. The plates were incubated at 37°C with saturated humidity while gently shaken by an orbital shaker (Polymax 1040, Heidolph Instruments GmbH, Schwabach, Germany) at 10 rpm. Every hour, samples of 100 μL were withdrawn from the acceptor compartment and replaced by the same volume of preheated buffer. 15 μL of 5 M NaOH were added to these samples to hydrolyze FDA to sodium fluorescein and the fluorescence intensity was measured at an excitation wavelength of 485 nm and an emission wavelength of 515 nm using a microplate reader (Tecan infinite, M200 spectrometer, Grödig, Austria). The amount of SEDDS that diffused through the mucus was calculated as percentage of a 100% control value, which was measured with the same transwell setup without mucus. With this control, also a potential influence of the ThinCert™ membrane could be excluded.

#### Rotating Tubes

The diffusion of SEDDS through the mucus was furthermore investigated by the rotating tube technique (15). Therefore, 100 μL of mucus was filled in silicone tubes with an inner diameter of 3 mm and one end was closed with a silicone cap. Then, 50 μL of the samples (SEDDS emulsions of FDA labelled formulation #3, see section 2.5) were applied to the open end, respectively, and the tube was closed with a second cap. To the control tube, 50 μL of buffer without SEDDS and FDA were applied. The filled tubes were incubated at 37°C and rotated horizontally at 50 rpm. After an incubation time of 4 h, the tubes were frozen at −80°C overnight and subsequently cut into slices of 2 mm thickness. The slices were suspended in 300 μL of 5 M NaOH, sonicated and subsequently incubated at 37°C for 30 min to extract and hydrolyze FDA to sodium fluorescein. The fluorescence of the samples was measured at an excitation wavelength of 485 nm and an emission wavelength of 515 nm and the fluorescence of the control was subtracted.

### Statistical Data Analysis

Statistical data analysis was performed with GraphPad Prism® 5. One-way ANOVA in combination with Bonferroni post test was used to calculate the statistical significance.

## Results

### Characterization of Stearic Acid Phosphotyrosine Amide

**Fig. 1:**
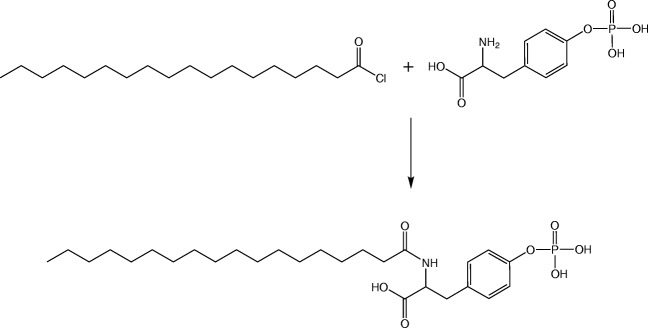
Scheme of synthesis of stearic acid phosphotyrosine amide (SA-PTyr). Stearoyl chloride was conjugated with phosphotyrosine in a nucleophilic acyl substitution

The synthesis of the stearic acid phosphotyrosine amide is shown in Fig. 1. Stearoyl chloride was conjugated with phosphotyrosine in a nucleophilic acyl substitution by forming a covalent amide bond between the amino group of the phosphotyrosine and the carbonyl carbon of the stearoyl chloride. After the precipitation of the product, the excessive unconjugated phosphotyrosine was elutriated by repeated washing steps. A quantification of the amount of conjugated phosphotyrosine was performed spectrophotometrically and a reaction yield of 62.1 ± 4.0% was determined.

In a qualitative analysis by mass spectrometry, a molecular ion peak at 527.6331 g/mol confirmed the successful formation of the derivative stearic acid phosphotyrosine amide. The HLB value of the derivative was calculated to be 10.9.

### Preparation and Characterization of SEDDS

Multiple SEDDS formulations composed of different ratios and combinations of stearic acid phosphotyrosine amide, Capmul MCM, TWEEN 80 and PEG 400 were prepared (Table 1). The mixing of the excipients resulted in homogenous preconcentrates. In order to investigate the properties of the SEDDS, emulsions with a concentration of 1% (*v*/v) were prepared. The originating SEDDS emulsions formed within less than 1 min and appeared to be clear to slightly bluish transparent emulsions.

#### Zeta Potential Change

**Fig. 2:**
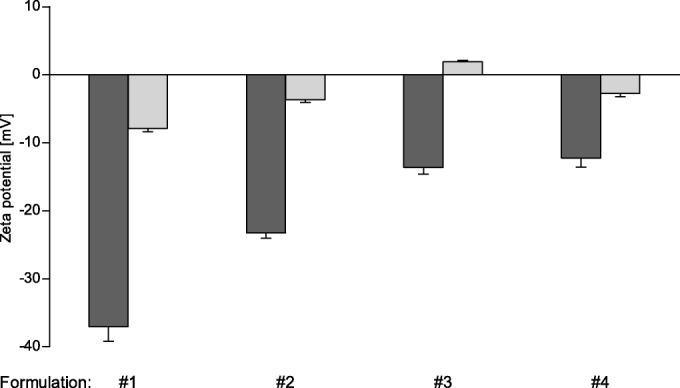
Change of zeta potential: Bars show the zeta potential of different SEDDS formulations before (dark gray bars) and after (light gray bars) enzymatic cleavage by intestinal alkaline phosphatase. Indicated values are the means of three experiments ± standard deviation. Zeta potentials before and after enzymatic cleavage differ significantly for each formulation, respectively (*p* < 0.0001)

**Fig. 3:**
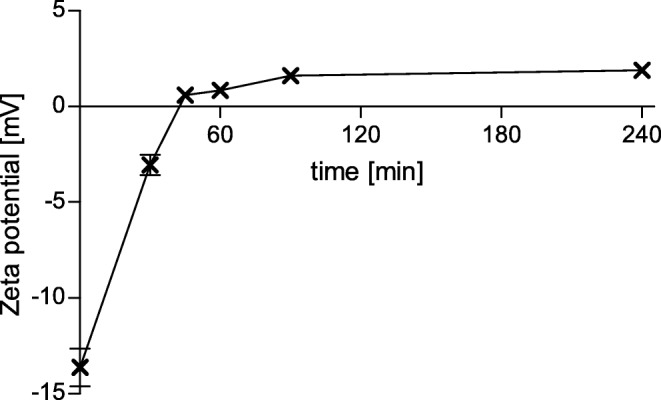
Kinetics of zeta potential change: The graph shows the zeta potential of SEDDS formulation #3, measured at pre-determined time points during incubation with IAP. Indicated values are the means of three experiments ± standard deviation

The SEDDS emulsions were incubated with IAP in order to induce a zeta potential change by enzymatic cleavage within the phosphotyrosine side chain. The zeta potential before and after incubation with the enzyme was measured by electrophoretic light scattering. A comparison of the zeta potential before and after the incubation with IAP is depicted in Fig. 2. For all tested SEDDS emulsions a change to a higher zeta potential after enzymatic cleavage compared to the undigested SEDDS emulsions could be observed. For formulation #3 a change from a negative zeta potential of −13.6 mV to a positive zeta potential of 1.9 mV was measured. Therefore, this formulation was chosen for further investigations: the kinetics of the change of zeta potential of formulation #3 over time is illustrated in Fig. 3. The change to a positive zeta potential occurred already between 30 to 45 min and after this, the positive zeta potential slowly further increased. After approximately 90 min a plateau was reached and no further significant change of the zeta potential until the last measured time point at 240 min was observed.

By means of dynamic light scattering, the droplet size of the SEDDS emulsion of formulation #3 was determined to be 66.1 ± 0.8 nm (at 37°C).

### Experiments on Caco-2 Cell Monolayers

**Fig. 4:**
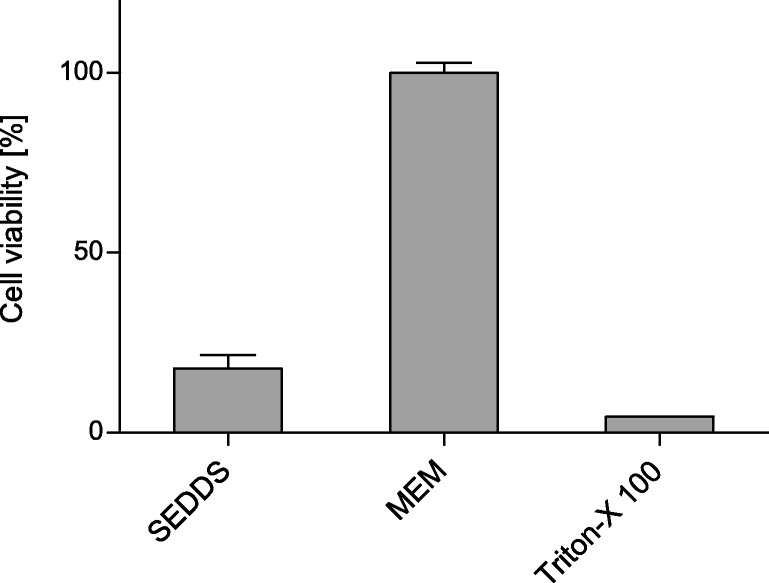
Influence of 1% (*v*/v) SEDDS formulation on cell viability of Caco-2 cells by means of a resazurin assay after incubation of 6 h. Indicated values are means of three independent experiments ± standard deviation

**Fig. 5:**
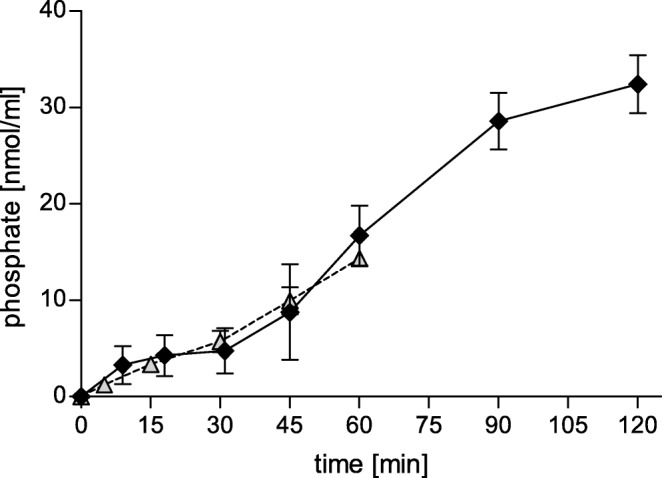
Time dependent phosphate release. SEDDS formulations were incubated on a Caco-2 monolayer and the released phosphate over 120 min after enzymatic cleavage was quantified (black rhombi). For comparison, the released phosphate after incubation with IAP for 60 min is indicated (light gray triangles). Indicated values are the means of at least three experiments ± standard deviation

The viability of Caco-2 cells after incubation with 1% (*v*/v) SEDDS emulsion was evaluated with a resazurin assay. As depicted in Fig. 4, after 6 h of incubation a notable decrease of viability was determined. The enzymatic cleavage of phosphate from the SEDDS by Caco-2 cells was investigated. The phosphate release over time is represented graphically in Fig. 5. The kinetics of the release is comparable to release of phosphate by isolated IAP (data not shown).

### Mucus Diffusion Studies

**Fig. 6:**
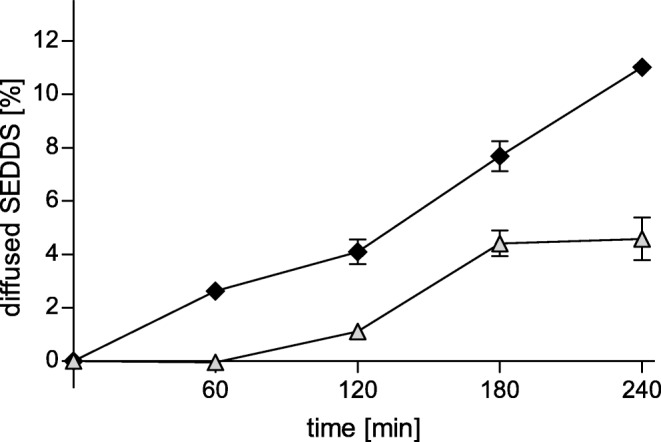
Results of mucus diffusion study with transwell chamber setup: SEDDS were loaded in the donor compartment and diffused through the mucus into the acceptor compartment. The percentage of SEDDS detected in the acceptor compartment of SEDDS before (black rhombi) and after (light gray triangles) zeta potential change at pre-determined time points is illustrated. Indicated values are the means of at least three experiments ± standard deviation

Mucus diffusion studies were performed with formulation #3, in order to compare the mucus diffusion of the FDA-labelled SEDDS before and after the change of zeta potential. The change was induced by pre-treatment with IAP prior to the experiment. With transwell chambers and rotating tubes, two different mucus diffusion test setups were employed. The results of the transwell chamber setup are depicted in Fig. 6, showing significant differences in the amounts of diffused SEDDS for all determined points of time. After a time period of 240 min, the amount of pretreated SEDDS detected in the acceptor compartment was 2.4-fold lower than the amount of untreated SEDDS.

**Fig. 7:**
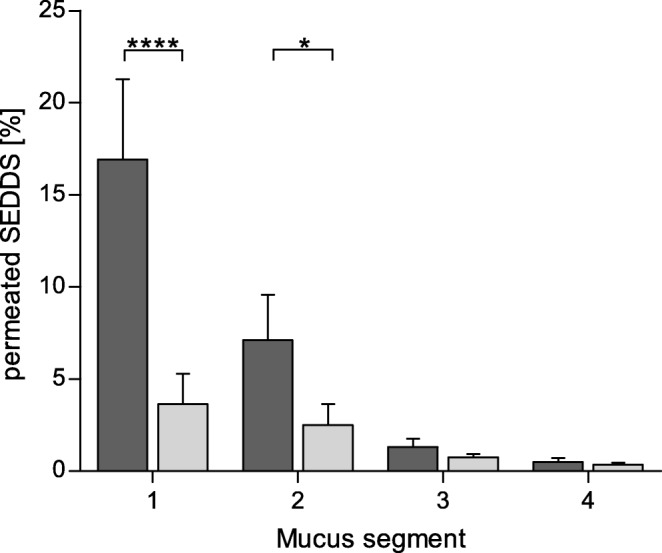
Results of mucus diffusion study with the rotating tube setup: Each mucus segment (1–4) represents a mucus layer of 2 mm thickness inside the silicone tube (1: 0–2 mm, 2: 2–4 mm; 3: 4–6 mm; 4: 6–8 mm). SEDDS were loaded in front of segment 1 and diffused into the mucus. The percentage of SEDDS detected in the respective mucus segment is shown for SEDDS before (dark gray bars) and after (light gray bars) zeta potential change. Indicated values are the means of at least three experiments ± standard deviation. Amounts of diffused SEDDS before and after zeta potential change differ significantly in segments 1 and 2, respectively (****p < 0.0001, *p < 0.05)

These findings are supported by the results of the mucus diffusion study using the rotating tube technique, which are shown in Fig. 7. The percentage of SEDDS pretreated with IAP that diffused into the mucus layer was significantly lower than that of untreated SEDDS. This is in particular evident for slices 1 and 2 with an up to 4.7-fold difference. The total accumulated amount of SEDDS that penetrated into the mucus layer (slices 1–4) was 7.6% of the pretreated SEDDS compared to 27.4% of the untreated SEDDS (3.6-fold difference).

## Discussion

In recent years, the number of protein and gene drugs has vastly increased and with constant progressions in understanding of diseases and technologies of drug design a further growth of this pharmaceutical sector can be expected. Currently, most of these drugs have to be applied parenterally due to a very limited bioavailability after oral administration. However, zeta potential changing drug delivery systems could be a solution and different approaches to this concept have been evaluated in recent publications (4–10).

In the present study, PTyr was conjugated with stearoyl chloride to create a molecule with amphiphilic properties and presenting a monophosphate on its polar head group. Due to its amphiphilic properties, the newly derivatized molecule stearic acid phosphotyrosine amide (SA-PTyr) can be implemented in SEDDS, presenting the PTyr on the outer surface. PTyr is substrate to intestinal alkaline phosphate - an enzyme, which is expressed and secreted by epithelial cells in the gastro intestinal tract and hydrolyzes the monophosphate ester present in the PTyr.

SEDDS formulations containing different amounts of SA-PTyr as well as Capmul MCM, TWEEN 80 were prepared (Table 1). For all following experiments, 1% (*V*/V) emulsions were prepared. This comparatively high concentration was applied for analytical reasons. In vivo, however, much lower concentrations in the range of 0.05% to 0.2% are expected (7).

A negative zeta potential was measured for all formulations, ranging from −37 mV to −12 mV (Fig. 2). With the monophosphate on the surface, the SEDDS show a negative zeta potential, which was the more negative the more SA-PTyr was incorporated in the SEDDS.

Subsequently, the zeta potential change after incubation with the enzyme IAP was investigated: for all investigated formulations, a change of zeta potential after incubation with IAP for 2 h could be measured (Fig. 2). The IAP hydrolyzes the monophosphate on the surface of the SEDDS and thus the negatively charged phosphotyrosine is converted to the neutral tyrosine. Accordingly, the amount of negatively charged groups on the surface of the SEDDS is reduced, which effects the change of the zeta potential. The more SA-PTyr present in the formulation and thus the more phosphate esters available for hydrolysis, the higher was the shift of the zeta potential. In case of formulation #3, the negative zeta potential of −13.6 mV (untreated) was changed to a positive zeta potential of 1.9 mV. Although the zeta potential of formulation #4 was less negative before incubation with IAP, other than formulation #3 it did not change to a positive zeta potential. This indicates that not enough PTyr was present to be cleaved by the IAP. In contrast, in formulations #1 and #2 too much PTyr seemed to be present, as the zeta potential remained negative, although the absolute change was higher. It can be assumed that not all PTyr could be cleaved. Based on these results, formulation #3, showing most promising properties, was chosen for further evaluations.

In an evaluation of the kinetics of the zeta potential change, a positive zeta potential was measured already after 45 min, slowly further increasing thereafter and reaching a plateau at around 90 min (Fig. 3). No further significant change of the zeta potential occurred until 240 min (last measured time point), which confirmed the stability of the emulsion and zeta potential of the droplets. This relatively fast change of the zeta potential is important, as, according to literature, the mucus turnover time in the human GIT is 4–6 h (16). Therefore, a fast change to a positive zeta potential is desirable, to give enough time for cell uptake of the positively charged SEDDS, once immobilized in the mucus layer due to interactions with the negatively charged mucus. As it is a brush border enzyme, the activity of the IAP is the highest close to the epithelium (17), which is why it can be expected that the cleavage and thus the change will mainly take place close to the epithelial cells, as desired.

The SEDDS were incubated on a Caco-2 monolayer in order to show that the incorporated SA-PTyr can not only be cleaved by isolated IAP, but also by IAP expressed by cells. In order to do so, first a cell viability test was performed. The resazurin assay showed a notable reduction of cell viability after an incubation time of 6 h. This can be explained by the relatively high concentration of the 1% (*v*/v) SEDDS emulsion, as it is known that surfactants show toxic effects at higher concentrations (18,19). The concentration of a SEDDS emulsion needed for drug delivery is expected to be much lower and thus the toxic profile of the highly concentrated SEDDS emulsion is not considered an issue. It has already been shown in several studies that SEDDS show a concentration dependent toxicity and do not have a negative effect on cell viability in concentrations ≤0.2% (7,19–22) being of relevance for drug delivery. However, for this experiment the high concentration was chosen in order to gain detectable results in the following phosphate release experiment. The incubation time for this experiment was therefore reduced to 2 h to avoid an influence of the cell toxicity. The result of the enzymatic cleavage investigation showed a successful cleavage of the phosphate over time. This result indicates that no steric hindrance prevents the enzyme from binding to the substrate.

With the purpose of comparing the mucus diffusion capabilities of SEDDS formulation #3 before and after the change of zeta potential, mucus diffusion studies were performed. To facilitate an easy detection of SEDDS, they were labelled with FDA. Due to its lipophilic properties, FDA can be incorporated in SEDDS and used as sensitive marker (10,14,23,24). A clear correlation between the change of zeta potential and the mucus diffusion strength could be determined. For the SEDDS that were pretreated with IAP and thus displayed a positive zeta potential, a significantly lower mucus diffusion compared to untreated, negatively charged SEDDS, was observed, confirming that a negative charge of the SEDDS favors the mucus diffusing capabilities and a positive charge leads to an immobilization of the SEDDS. This difference could be shown with both mucus diffusion setups that were evaluated:

In the transwell chamber setup, SEDDS emulsions were applied on top of a mucus layer in a transwell insert and their diffusion through the mucus into the acceptor compartment was investigated. It was shown that the negatively charged SEDDS diffuse significantly faster through the mucus layer than the positively charged SEDDS, which had been treated with the IAP before the experiment. After the first hour even only negatively charged SEDDS were detected to have crossed the mucus layer. As mentioned above, the mucus turnover time in the human GIT is approximately 4–6 h (16). Therefore, a fast diffusion into the mucus layer is essential in order to facilitate the cell uptake of the SEDDS before the mucus turnover transport the SEDDS away.

In the rotating tube setup, SEDDS emulsions were loaded in front of the mucus in a silicone tube and their diffusion into the mucus was investigated. In total, 3.6-fold more negatively charged SEDDS diffused into the mucus compared to the positively charged SEDDS and this difference was most pronounced in the first two of four segments. Considering that the mucus layer present on the epithelium of the human GIT has a thickness between 10 and 200 μm (16,25,26), these first segments can be seen as the most representative.

Related to the mucus layer present on the epithelium of the human GIT this means that the applied SEDDS with their negative zeta potential will diffuse through the mucus layer. Once their zeta potential changes in succession to the enzymatic cleavage by the intestinal alkaline phosphatase secreted by the epithelial cells and the resulting zeta potential change to positive, they will be immobilized close to the epithelium. The immobilization supports an enhanced uptake probability due to the increased residence time and the positive charge of the SEDDS enhances the cell uptake as a result of electrostatic interactions of the positively charged SEDDS with the negatively charged cell membrane (11,12).

## Conclusion

Stearoyl chloride was successfully conjugated with phosphotyrosine. The resulting compound stearic acid phosphotyrosine amide was incorporated into SEDDS, creating a negative zeta potential. For SEDDS formulation #3 a zeta potential change after enzymatic cleavage from −13.6 mV to +1.9 mV could be demonstrated. In two different mucus diffusion studies significant differences in the mucus diffusion strength of SEDDS formulation #3 before and after zeta potential change were shown. These findings suggest that stearic acid phosphotyrosine amide is a promising excipient for the preparation of zeta potential changing SEDDS, which show good mucus diffusion properties before and good immobilization in the mucus after enzymatically induced zeta potential change.

## Abbreviations

DMSO: Dimethyl sulfoxide
FDA: Fluorescein diacetate
GIT: Gastrointestinal tract
HBS: HEPES-buffered Saline
IAP: Intestinal alkaline phosphatase
MEM: Minimum essential medium
PBS: Phosphate-buffered saline
PTyr: Phosphotyrosine
SA-PTyr: Stearic acid phosphotyrosine amide
SEDDS: Self-emulsifying drug delivery system
